# Research hotspots and trends of chordoma: A bibliometric analysis

**DOI:** 10.3389/fonc.2022.946597

**Published:** 2022-09-16

**Authors:** Jianxuan Gao, Runzhi Huang, Huabin Yin, Dianwen Song, Tong Meng

**Affiliations:** ^1^ Department of Spine Surgery, Shanghai General Hospital, School of Medicine, Shanghai Jiaotong University, Shanghai, China; ^2^ Tongji University Cancer Center, Shanghai Tenth People’s Hospital, School of Medicine, Tongji University, Shanghai, China; ^3^ Department of Spine Surgery, Tongji Hospital, Tongji University School of Medicine, Tongji University, Shanghai, China

**Keywords:** chordoma, bibliometric analysis, hotspots, treatment, tumorigenesis

## Abstract

**Background:**

Chordoma is a type of mesenchymal malignancy with a high recurrence rate and poor prognosis. Due to its rarity, the tumorigenic mechanism and optimal therapeutic strategy are not well known.

**Methods:**

All relevant articles of chordoma research from 1 January 2000 to 26 April 2022 were obtained from Web of Science Core Collection database. Blibliometrix was used to acquire basic publication data. Visualization and data table of collaboration network, dynamic analysis, trend topics, thematic map, and factorial analysis were acquired using Blibliometrix package. VOSviewer was used to generate a visualization map of co-citation analysis and co-occurrence.

**Results:**

A total of 2,285 articles related to chordoma were identified. The most influential and productive country/region was the United States, and Capital Medical University has published the most articles. Among all high-impact authors, Adrienne M. Flanagan had the highest average citation rate. Neurosurgery was the important periodical for chordoma research with the highest total/average citation rate. We focused on four hotspots in recent chordoma research. The research on surgical treatment and radiotherapy was relatively mature. The molecular signaling pathway, targeted therapy and immunotherapy for chordoma are not yet mature, which will be the future trends of chordoma research.

**Conclusion:**

This study indicates that chordoma studies are increasing. Surgery and radiotherapy are well reported and always play fundamental roles in chordoma treatment. The molecular signaling pathway, targeted therapy, and immunotherapy of chordoma are the latest research hotspots.

## Introduction

Chordoma is a relatively rare malignancy characterized by local invasion (1). To reduce the risk of recurrence and improve the prognosis of patients, the en-bloc tumor resection with wide margins is recommended (2). Due to the notochord origination, chordoma is normally located in the axial skeleton, such as the skull base and sacrum (3). These specific anatomical structures compromise the application of en-bloc methods, leading to a high relapse rate (4, 5) . In addition, the chemo-/radiotherapy resistance features also challenge the management of chordoma (6). To improve the prognosis of chordoma patients, exploring the tumorigenic mechanisms and optimizing therapeutic strategies are pressing needs.

Pathologically, chordoma is derived from notochord remnants with the impacts of carcinogenic factors. Therefore, the identification of tumorigenic biomarkers may provide therapeutic targets for chordoma (7). Receptor tyrosine kinases (RTKs), which are crucial regulators of chordoma, can activate signaling cascades, leading to the dysfunction of various essential proteins. Thus, tyrosine kinase inhibitors (TKIs) are widely used in clinical practice for chordoma, such as imatinib, sunitinib, and apatinib (8, 9). Besides targeted therapies, novel techniques in adjuvant radiotherapies like proton and carbon ion therapy have been applied in chordoma with promising therapeutic effects (10, 11). Thus, recent progresses of basic and clinical research optimize chordoma treatment and improve patients’ prognosis.

Due to the rarity of chordoma, studies focused on this malignancy are still limited. Given this limitation, it would be useful to summarize the current hotspots and future trends of chordoma research. Bibliometrics, a quantitative and qualitative analysis, can assist researchers in refining current research hotspots and future development trends by co-word and co-citation (12). Recently, bibliometrics and visualization have been used to analyze various fields of research, such as coronavirus disease 2019 (COVID-19) and breast cancer, whereas none focus on chordoma (13–15). In this study, we collected and collated the publications on chordoma in the twenty-first century from the Web of Science (Wos) database. By analyzing the current research hotspots and knowledge framework in chordoma field, we point out its research emphases and future trends.

## Methods

### Data sources and retrieval strategies

This study was approved by our Institutional Review Board (IRB). Data were obtained from the Wos Core Collection and analyzed by bibliometric analysis software. Publications were selected from SSCI and SCIE indexes, excluding other databases, such as Scopus. The retrieval strategy was as follows: subject words= chordoma or chordomas, literature type= article, language= English, date= 1 January 2000–26 April 2022. A total of 2,285 studies were retrieved. All records and references were downloaded in a TXT format, and all literature retrievals and data extractions were introduced into VOSviewer software (version 1.6.18) and R software version 4.1.0 using Bibliometrix R package (version 3.2.1) and Biblioshiny for further analysis.

### Data and statistical analysis

Biblimetrix package is an open-source tool to analyze publications by qualitative and quantitative method (16). Biblimetrix package could convert and output simplify bibliographic information and complete data analysis and visualization, including annual scientific production, collaboration network, dynamic analysis, trend topics, thematic map, and factorial analysis. The institution impact or author impact was mainly determined by h-index, which was used to quantify the certain institutions and authors scientific influence by statistics of publication number and citation frequency (17).

Vosviewer was used to analyze bibliographic data on countries, institutions, authors, citations, and keywords and construct network maps of co-authorship, co-citation, and co-occurrence (18). In addition, VOSviewer could output three different visualization maps by setting thresholds: network visualization, overlay visualization, and density visualization.

## Results

### Annual publication analysis

**Figure 1:**
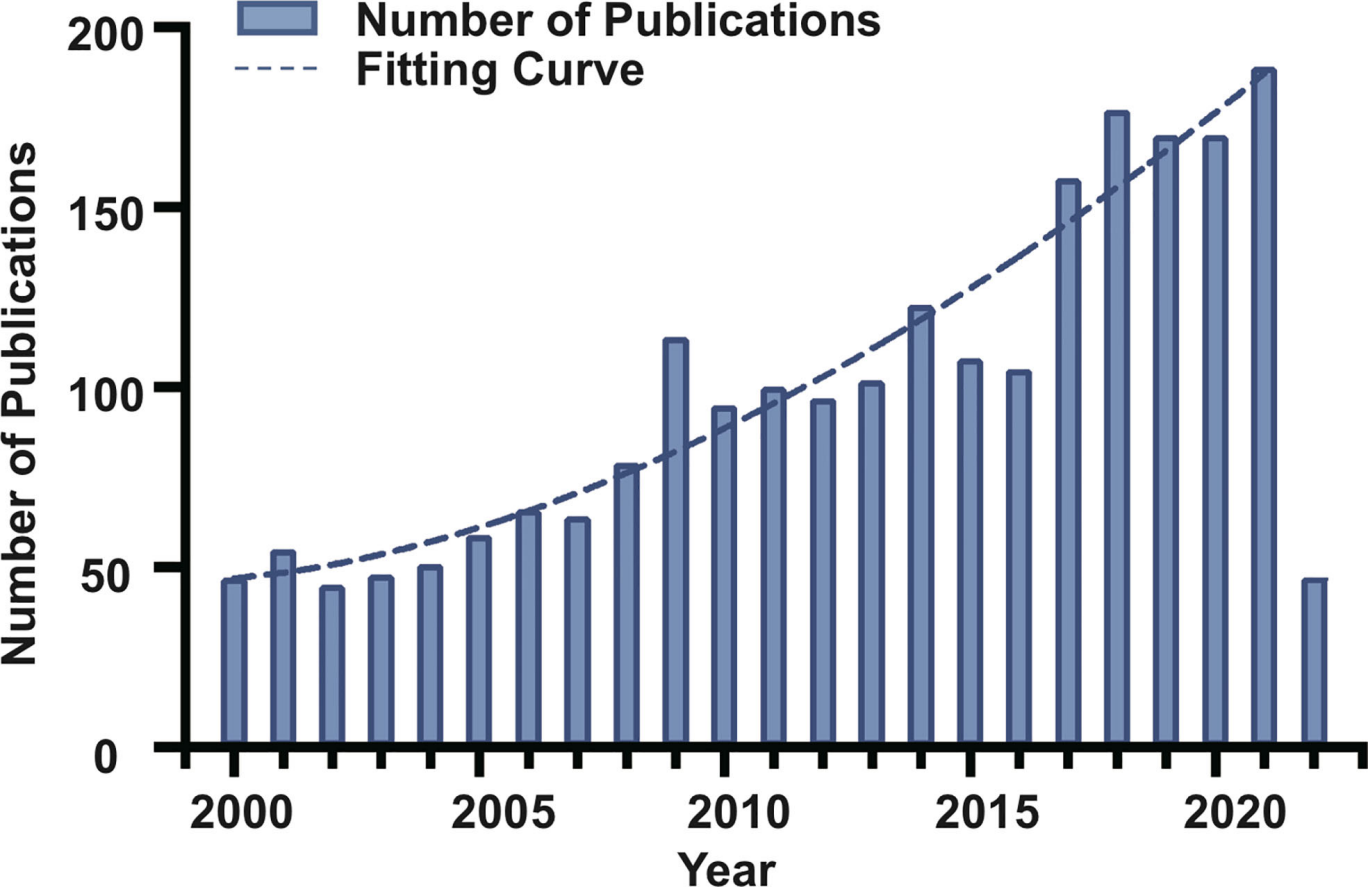
Trend of annual publication numbers and fitting curve for chordoma research in the twenty-first century.

A total of 2,285 articles were published in the chordoma field with a total citation frequency of 42,808 times (**Table 1**). The fitting curve of annual publication growth trend was y $\frac{102,808.8}{1+(\frac{x}{981.7}{)}^{-1.738}}+46.20$ =( R2= 0.9244), consistent with the current research trend of chordoma, so was the curve of annual cited frequency growth trend (**Figure 1**; **Supplementary Figure S1**). Since the twenty-first century, more scientific issues have been focused on chordoma. The research progress of chordoma has emerged endlessly, and the annual numbers of articles are also increasing year by year, indicating that chordoma is attracting more attention, and chordoma research has great clinical significance and development potential.

**Table 1:** Main information of publications.

Description	Results
Timespan	2000.01.01-2022.04.26
Sources (journals, books, etc)	594
Documents (articles)	2,285
Times Cited	42,808
References	31,268
Countries	68
Institutions	2,267
Funds	781
Authors	9,716
All Keywords	6,070
Author Keywords	3,691

### Most productive countries/regions and institutions

**Figure 2:**
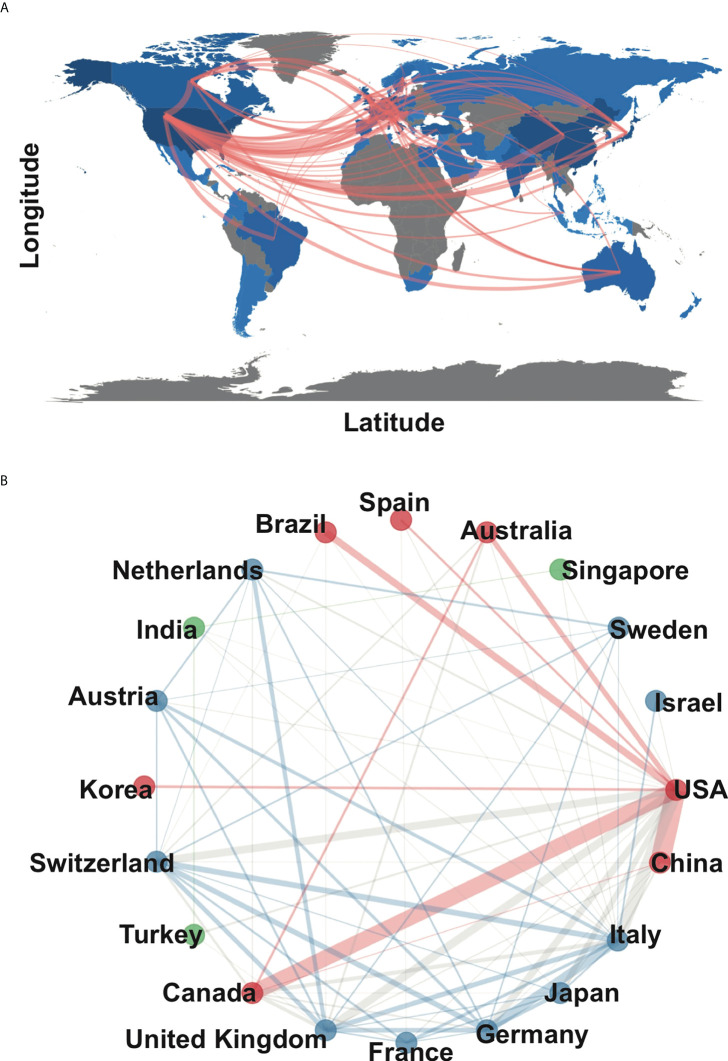
Main countries/regions of chordoma research and collaboration. **(A)** Countries/regions distribution of chordoma research and collaboration map; **(B)** collaboration network of 20 countries/regions in chordoma research.

Since 2000, 68 countries/regions participated in chordoma research, of which the top 10 cumulative publication frequency was 1,880. Google Earth mapped the country/region distribution based on the number of publications (**Figure 2A**). Three countries published more than 1,000 articles, namely, the United States (N=703), China (N=306), and Japan (N=210) (**Supplementary Table S1**). Regarding the regional distribution of chordoma incidence rate, Asian/Pacific Islander (0.096; 95%CI 0.082–0.111) and White (0.093; 95%CI 0.093–0.096) individuals were relatively high, while American Indian/Alaskan Native (0.049; 95%CI 0.029–0.077) and Black (0.042; 95%CI 0.036–0.048) were correspondingly low (1). Thus, regional incidence distribution might be associated with country-related chordoma publications. Centrality is often used to evaluate the importance of nodes in the network. The United States had the highest centrality, indicating its prolificacy and influence in chordoma research. The United States paid the best attention to regional cooperation among countries, especially China, which promotes the rapid construction of knowledge framework (**Figure 2B**). In addition, high-impact institutions/authors in various countries cooperated with those in the United States inordinately (**Supplementary Figures S2A, B**). Collectively, we supposed that besides the incidence rate of chordoma, regional cooperation is another crucial driving force for chordoma research development.

A total of 2,098 institutions were involved in chordoma research (**Supplementary Table S2**), in which Capital Medical University has published the most articles (N=126), followed by University of Pittsburgh (N=124) and Massachusetts General Hospital (N=122). Among the top 10 institutions with the most article productions, seven are from the United States, publishing 666 articles with an average citation frequency of 23.9 times. The other three are from China (N=126, TC=696), Austria (N=87, TC=510), and Canada (N=66, TC=1,029), respectively. In recent years, Chinese institutions ranked high in total publication volume, whereas the h-index and average citations were lower than most institutions in the United States.

Fund is an important driving force for institutions to carry out research progress. Since 2000, 781 different types of funds financially aided chordoma research worldwide. Projects funded by US National Institutes of Health and United States Department of Health and Human Services produced the same number of articles (N=142), followed by National Natural Science Foundation of China (N=112). In the top 10 funds, four are from the United States, with three from China, two from Japan, and one from Europe (**Supplementary Table S3**). Based on these 10 funds, 625 articles were published, accounting for 27.4% of all studies. In addition, Chordoma Foundation, a non-profit organization, also plays a pivotal role in advancing chordoma research. Composed of more than 400 chordoma specialists worldwide, Chordoma Foundation provides trusted resources and assistance to thousands of researchers and patients around the world. By 2022, Chordoma Foundation has funded 28 studies and supported 73 chordoma-related studies in various forms. Therefore, institutions’ prolificacy and high impact were related to the national and fund support for chordoma research.

### Most productive and influential journal and author analysis

#### Journal analysis

We identified 594 journals publishing articles focused on chordoma. The top 10 published 551 articles accounted for 24.1% of all articles. World Neurosurgery, Journal of Neurosurgery, Neurosurgery, Spine, and Journal of Neurosurgery-Spine are the top 5 productive journals with Nature Medicine, Nature Genetics, European Urology, Acta Neuropathologica, and Journal of the American Chemical Society as top 5 high-impact journals (**Table 2**; **Supplementary Table S4**).

**Table 2:** Top 10 most productive journals in chordoma field.

Rank	Journal	Records	Proportion (%)	Total Citations	Citations per item	h-index
1	World Neurosurgery	98	4.29	885	9.03	18
2	Journal of Neurosurgery	77	3.37	2,251	29.23	27
3	Neurosurgery	66	2.89	3,818	57.85	33
4	Spine	60	2.63	2,028	33.80	24
5	Journal of Neurosurgery-Spine	52	2.28	1,477	28.40	21
6	International Journal of Radiation Oncology Biology Physics	47	2.06	2,450	52.13	29
7	Acta Neurochirurgica	46	2.04	644	14	15
8	European Spine Journal	41	1.79	804	19.61	18
9	Chordomas and Chondrosarcomas of the Skull Base and Spine	33	1.44	11	0.33	1
10	Journal of Clinical Neuroscience	31	1.37	333	10.74	11

Total/average citations indicate the quality and impact, in which Neurosurgery ranked the first place. Its research topics of chordoma ranged from surgical options to combined treatment of surgery and adjuvant therapy. The recent topics are multi-dimensional therapeutic strategies including targeted therapy and immunotherapy, while scholars paid more attention to recurrent chordoma treatment. Chordoma articles in high-impact journals are mostly associated with multi-omics sequencing and clinical trials to identify tumorigenic signaling pathways, therapeutic targets, and novel treatment strategy.

**Figure 3:**
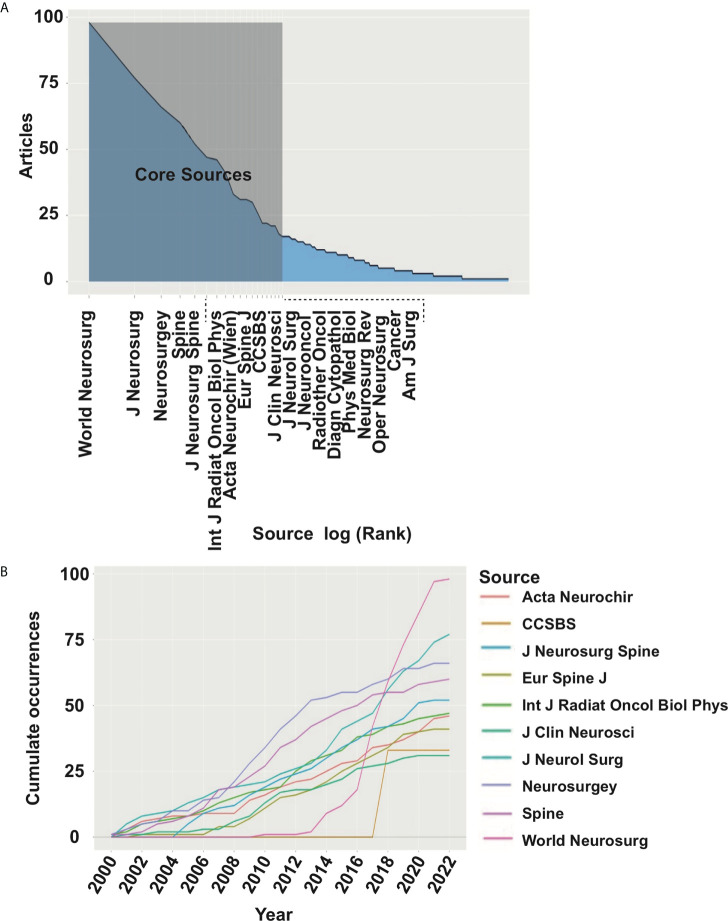
Core journals in chordoma research. **(A)** Identification of 19 core journals of chordoma field by law of Bradford; **(B)** publication numbers of top 10 core journal dynamic trend.

Currently, the research system of the chordoma field is relatively mature, and the distribution and number of core journals accord with law of Bradford. The number of articles published in these journals shows an upward trend year by year (**Figures 3A, B**). As different journals have preferences for specific research fields, to grasp the hotspots of chordoma, we compared the changes in journals focusing on chordoma. The results revealed that studies of novel surgery and radiotherapy options, immune microenvironment, and tumorigenic signaling pathways are gradually expanding.

### Most productive and influential authors

A total of 9,716 authors published articles in the chordoma field. As the first/corresponding author, Junting Zhang (N=21), Huilin Yang (N=20), and Yazhuo Zhang (N=19) are the top 3 high-yield authors (**Supplementary Table S5**). H-index of articles from Huilin Yang was 10, which ranked first among these authors. In addition to productivity, the average citation rate also optimizes the assessment of scientific influence. The average citation rate of Adrienne M. Flanagan was as high as 80.36. Adrienne M. Flanagan made significant contributions to the histopathology, tumorigenic mechanism of chordoma, and identification of potential therapeutic targets. Based on the law of Lotka, a relatively stable author-cooperation group has formed in chordoma fields, which cultivated several well-known scholars to promote the development of chordoma research (**Supplementary Figures S3A, B**).

### Research hotspots and trends

#### Co-citation analysis

**Figure 4:**
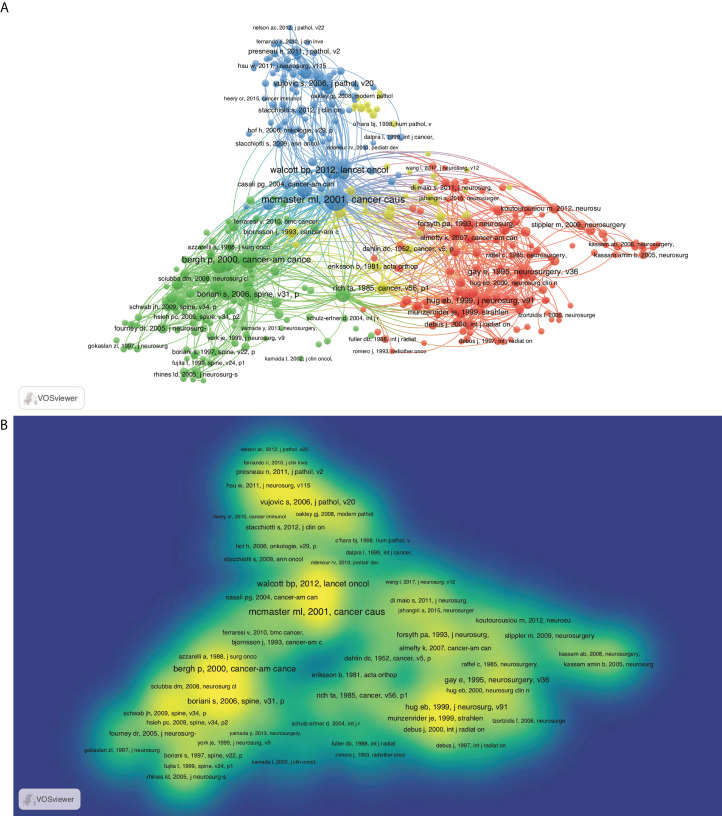
Co-citation map based on VOSviewer in chordoma field. **(A)** Network visualization; **(B)** density visualization.

Citation is a useful method to evaluate the impact and recognition in the scientific community. Thus, identification of highly cited articles assists in recognizing the hotspots and future trends. All articles cited in chordoma publications were identified by co-citation analysis of VOSviewer (**Figure 4A**). Based on their intrinsic relevance, articles are divided into three color-coded clusters, in which the topics of blue cluster are oncogenic mechanism and targeted therapy; red and green are radiotherapy and surgery, respectively. The red/green nodes are distributed densely, while blue nodes are less. In the hotspots of chordoma research, the field of surgery and radiotherapy are relatively mature, while tumorigenic mechanism and targeted therapy are still in the development stage (**Figure 4B**).

In view of time-accumulating factors, the average citation rate per year was also adopted. The top 100 most-cited articles on average by year were analyzed, and the top 20 ones were described in **Supplementary Table S6**. The most cited article was an epidemiological survey published by McMaster et al., which was also the largest case series of chordoma in the United States before publication (3). This study analyzed and interpreted data on incidence, treatment, and survival patterns chordoma from 1973 to 1995 based on the Surveillance, Epidemiology, and End Results (SEER) program of the National Cancer Institute. Among them, surgical treatment and radiotherapy remained the hottest topics. To grasp the future trends, we ranked the 100 high-impact articles according to the publication date. In the past 5 years, most publications focused on tumorigenic signaling pathways, immune microenvironments, and targeted therapy for chordoma (**Supplementary Figure S4**), consistent with the above co-citation analysis. As the similar features of chordoma and chondrosarcoma, their treatment therapy and tumorigenic mechanism were discussed together previously. Currently, the multi-omics sequencing specific for chordoma has been performed, and several signaling pathways has been identified as candidate therapeutic targets.

#### Co-word analysis

**Figure 5:**
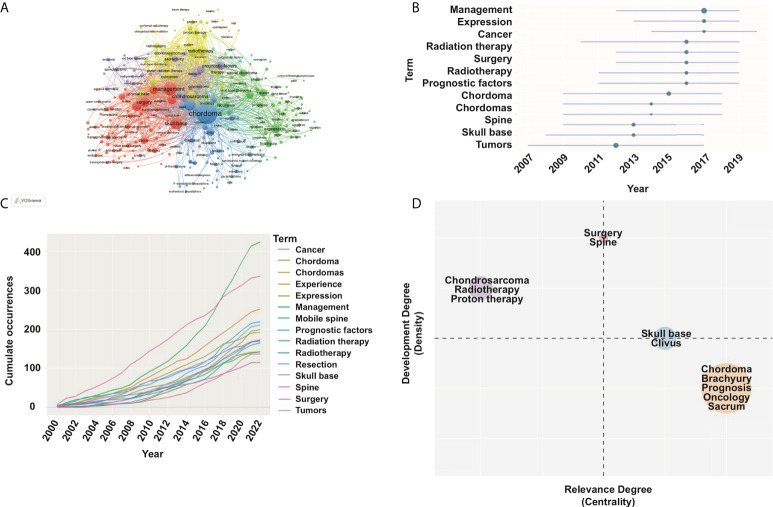
Keyword co-occurrence map with an occurrence frequency of more than 5 based on VOSviewer and Blibliometrix. **(A)** Network visualization; **(B)** topic trend of high-frequency keyword in chordoma filed; **(C)** high-frequency keyword dynamics; **(D)** strategic diagram in chordoma research.

Co-word analysis can effectively identify the high-frequency keywords and reveal the specific research topics, hotspots, and future trends. After literature search (chordoma-related publications, N=2,285; keywords, N=6,070), we analyzed the keywords provided by original authors and WoS using VOSviewer. Based on the frequency and ranking of keywords, we classified the 50 high-frequency keywords through co-occurrence network and Blibliometrix (**Figure 5A**; **Supplementary Figures S5A, B**). Based on the top 15 high-frequency keywords, surgery and radiotherapy are the focus of chordoma research. Recently, tumorigenic mechanism and molecular signaling pathway presented an upward trend (**Figures 5B, C**). Based on the strategic diagram analysis, we calculated four cluster’s centrality and density (**Figure 5D**). The cluster of surgery, located in the first quadrant, had high centrality and density. Thus, this topic is relatively mature and crucial for chordoma research. Besides, radiotherapy is also a mature research topic. Although the research of tumorigenic mechanism was in the central position of the chordoma field, the current research was still insufficient.

## Discussion

Chordoma is a relatively rare disease with a high relapse rate and poor prognosis. The tumorigenic mechanism and optimal therapeutic strategy are still unclear. In this article, we used bibliometrics to analyze the publications of chordoma since the twenty-first century. By 26 April 2022, a total of 2,285 articles in the field of chordoma have been published, with the citation times of 42,808. The most influential and productive country/region was the United States, followed by China and Japan. A total of 2,267 institutions participated in chordoma research, in which Capital Medical University published the most articles, followed by University of Pittsburgh and Massachusetts General Hospital. Among all high-impact authors, Huilin Yang had the highest h-index and Adrienne M. Flanagan had the highest citation rate and average citation rate. World Neurosurgery, Journal of Neurosurgery, Neurosurgery, Spine, and Journal of Neurosurgery-Spine were the first five periodicals with maximum publications in chordoma research. Among them, the highest total and average citation rate was Neurosurgery. Moreover, we also identified four hotspots by analyzing the topic of highly cited articles and co-word in the chordoma field: (1) molecular signaling pathway and targeted therapy of chordoma; (2) surgical treatment of chordoma; (3) optimization of radiotherapy; and (4) immunotherapy of chordoma.

### Molecular signaling pathway and targeted therapy of chordoma

Facing the treatment dilemma of chordoma, exploring the tumorigenic mechanisms and the corresponding molecular signaling pathways are important. TBXT gene is a transcription factor for notochord development, and its duplication confers major susceptibility to familial chordoma (19). Its encoding protein, brachyury, is highly expressed in chordomas, which are associated with progression-free survival of chordoma patients (5, 20, 21). Besides, other gene mutations have also been identified in chordoma, such as CDKN2A, TP53, and LYST (22). CDK4 and p53, which function in the G1 phase cell cycle, are related to overall survival (OS) of chordoma patients (23, 24). In an integrated multi-omics analysis, CA2 and THNSL2 were identified in the switched compartments, cell-specific boundaries, and loops, indicating their tumorigenic roles in chordoma (25). Based on their key roles in chordoma tumorigenesis, targeting them provides potential therapeutic options. CDK7/12/13 inhibitor and the CA2 inhibitor, Dorzolamide, have been found to inhibit chordoma *in vitro* and *in vivo* (20, 25).

RTKs, such as platelet-derived growth factor receptor (PDGFR), epidermal growth factor receptor (EGFR), fibroblast growth factor receptor (FGFR), and vascular endothelial growth factor receptor (VEGFR), are also important oncogenic regulators of chordoma (26–28). They continuously translate extracellular stimuli into intracellular signaling cascades, such as PI3K-Akt-mTOR, and promote cell growth and tumorigenicity (29–31). Imatinib, an inhibitor of PDGFR, was used in six patients with advanced chordoma, and patients’ prognoses were improved with more than 1 year follow-up (32). Gefitinib and afatinib, EGFR inhibitors, were also effective in chordoma patients (33, 34). With the treatment of apatinib, a potent inhibitor of VEGFR, one in seven patients achieved objective response according to RECIST and Choi criteria in 27 advanced chordoma patients (9). In addition, targeting PI3K-Akt-mTOR pathway worked well in TKI-resistant chordoma, and the combination of sirolimus plus imatinib significantly reduced the tumor size in imatinib-resistant chordoma patients (8, 35).

Chordoma in different locations also has variable biological behaviors and molecular features. Poorly differentiated chordoma is mainly found in the clivus/cervix location (36). Histologically, skull base chordoma had more abundant chondroid matrix and diffuse growth pattern, whereas sacral/spinal chordoma had non-chondroid, myxoid matrix, and a lobulating pattern (37). As for transcriptome biomarkers, LMX1A is dominant in skull base chordoma, and SALL3 is unique to spine chordoma (38). Additionally, compared to clival chordoma, higher cMET dependence was found in sacral chordoma, indicating that cMET inhibitors alone or in combination with other drugs might particularly benefit patients with sacral chordoma (27).

### Surgical treatment of chordoma

Surgical resection has always been a research hotspot in chordoma field and cornerstone of therapeutic strategy. The possible reasons for surgery as a key hotspot are as follows: (1) the complicated anatomical structures of axial skeleton and local invasiveness nature of chordoma increase the difficulty of resection (39, 40); (2) the surgical method is altered according to different locations (41–43); (3) the reconstruction is needed after tumor resection, and biomaterials with novel technologies, such as 3D printing, may provide optimized fixation (44); (4) the improvement of postoperative adjuvant therapy is beneficial to improve the survival outcomes of chordoma patients (45–47).

Chordoma-specific factors (volume, location, and pathological subtype) are associated with the surgical consideration, such as surgical method, approach, and scope. Take tumor location for example; skull base chordoma has a high recurrence rate because it infiltrates the skeleton and is closed to vital structures including brainstem and vital arteries. Thus, radical surgery combined with adjuvant radiotherapy is preferred for long-term survival (48). As for mobile spine and sacral chordoma, marginal resection or gross total resection is recommended (41). Based on single- and multi-center case series of chordoma, Meng et al. reported the glorious local control with the treatment of en-bloc spondylectomy (49, 50). However, more than 1 cm margin in three planes is hard to achieve even when performed by experienced surgeons (51). Therefore, accurate marginal excision is crucial, which promotes the application of computer navigation in tumor resection (52).

Bone stability maintenance is also a focus of tumor resection, and advanced biomaterials are widely used. Currently, custom-made prostheses have been successfully used, while 3D printing may have more benefits (44, 53). 3D-printed prostheses based on computer simulation can fit perfectly to defective sites and achieve personalized treatment (44, 54). Additionally, the bone contact surface of 3D-printed prosthesis can be made porous, inducing bone ingrowth to increase long-term stability (55). Other advanced biomaterial technologies, such as carbon-fiber-reinforced polyetheretherketone (CFR-PEEK) composite implants also improved the prognosis and decreased the risk of local recurrence (45).

### Optimization of radiotherapy

Conventional radiotherapy is almost ineffective for chordoma either alone or combined with surgery (56–58). As the specific anatomical structure of peritumoral spinal cord, nerve root, and vessels, the methods, timing and complications of radiotherapy have always been the research focus (56). Currently, proton and carbon ion therapies that relied on Bragg peak effects are the preferred radiotherapy in most quaternary centers. Proton and carbon ion radiotherapies have little damage to normal tissue and target chordoma site for maximum dose of radiation therapy (10, 11). Thus, they are effective treatment options for chordoma with acceptable radiation toxicity (59, 60). Timing of these high-dose radiotherapy is also taken into consideration. DeLaney et al. confirmed that survival outcome of chordoma patients was significantly improved with preoperative radiotherapy (61). However, the preoperative high-dose radiotherapy may increase the complication of wound healing, and primary resection combined with postoperative proton or carbon ion therapy was recommended by many researchers (62–64).

The duration, dosage, and adjuvant materials [e.g., phosphorus-32 (P32), yttrium] of proton or carbon ion therapy are also key points. Due to the radioresistant characteristic of chordoma, local control rate of low-dose radiotherapy is often poor. Therefore, proton or carbon ion therapy, whether as a radical therapy or adjuvant option, has high-dose requirements. Several studies reported that current dose of radiotherapy strategies typically exceeded 70 Gy, and multi-cycles therapy was necessary (56, 65, 66). The local recurrence rate could remain low under high-dose radiotherapy, but the radiotoxicity still needs further discussion. Folkert et al. reported dural plaques as an auxiliary mean of external irradiation could increase radiotherapy dose delivery in radiation-resistant chordoma and improve local control rates with less toxic risk (45, 67). Increasing radiotherapy sensitivity is another option to reduce the radiation resistance and complication. In a preclinical research, Hao Shuyu et al. identified a protein phosphatase 2A (PP2A) inhibitor LB100, which could serve as a supplement for radiation to effectively enhance DNA damage-induced chordoma cell death and delay tumor growth *in vivo (* 68).

### Immunotherapy of chordoma

Immune checkpoint can attenuate the tumor killing ability of T lymphocytes. Recently, immune checkpoint inhibitors worked well in the treatment of chordoma (69). Programmed cell death protein-1 (PD-1) and programmed cell death ligand-1 (PD-L1) are highly expressed in chordoma (70–72). Nivolumab, an anti-PD-1 monoclonal antibody, showed effectivity against chordoma *in vivo* and is currently evaluated in clinical trials for chordomas (73, 74). In addition, avelumab could inhibit the chordoma cell proliferation by targeting PD-L1, while further clinical evidence is still needed for chordoma (75).

As brachyury is highly expressed in chordoma, it may serve as a target of current immunotherapy. Brachyury vaccines have been produced. GI-6301, which delivers antigens through dendritic cells, specifically activates CD4^+^ and CD8^+^ cell and has a lethal effect on brachyury-expressing tumor cells (76, 77). Additionally, modified vaccinia Ankara (MVA) poxviral vaccine vector encodes human brachyury, and adenovirus serotype 5 (Ad5) has been developed and applied in clinical trials (5, 78). Thus, immunotherapy is regarded as one future trend in the basic research and clinical treatment of chordoma.

## Conclusion

This study indicates that chordoma studies are increasing. Surgery and radiotherapy are well reported and always play fundamental roles in chordoma treatment. The molecule signaling pathway, targeted therapy, and immunotherapy of chordoma are the latest research hotspots.

## Data availability statement

The original contributions presented in the study are included in the article/**Supplementary Material**. Further inquiries can be directed to the corresponding authors.

## Author contributions

TM conceived the project. JG, RH performed bioinformatics analysis. HY, DS and TM interpreted and analyzed data. JG and TM wrote the manuscript with comments from all authors. All authors contributed to the article and approved the submitted version.

## Funding

This work was supported in part by the National Natural Science Foundation of China (82173168), Postdoctoral Research Foundation of China (2021M702485), Shanghai Rising-Star Program (21QA1407500).

## Conflict of interest

The authors declare that the research was conducted in the absence of any commercial or financial relationships that could be construed as a potential conflict of interest.

## Publisher’s note

All claims expressed in this article are solely those of the authors and do not necessarily represent those of their affiliated organizations, or those of the publisher, the editors and the reviewers. Any product that may be evaluated in this article, or claim that may be made by its manufacturer, is not guaranteed or endorsed by the publisher.
