# Supplementary figures and images for: Research hotspots and trends of chordoma: A bibliometric analysis
Source: Front Oncol. 2022 Sep 16;12:946597. doi: 10.3389/fonc.2022.946597 (PMC9523362; doi:10.3389/fonc.2022.946597)

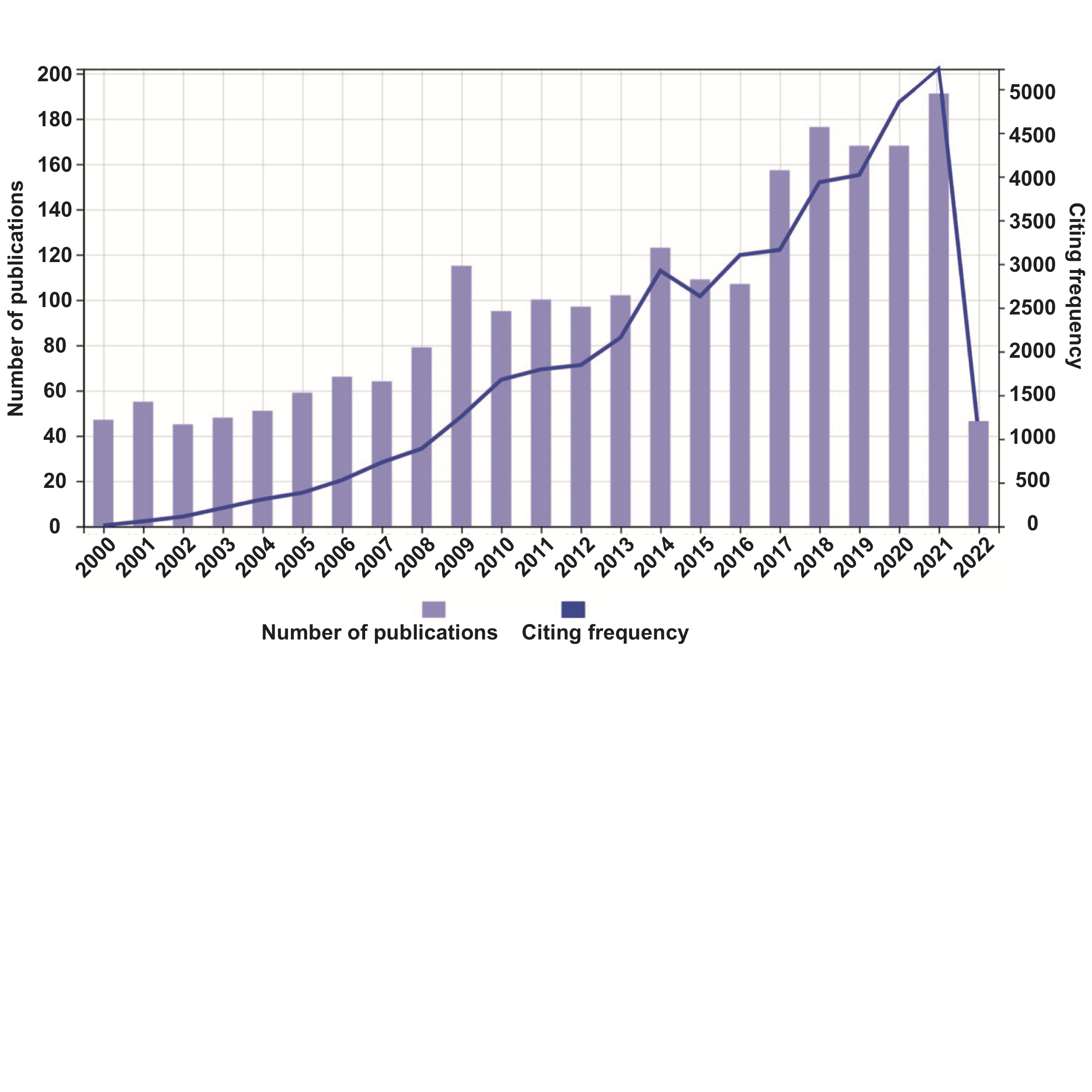

Supplement: Supplementary file 1 [file Image_1.tiff]

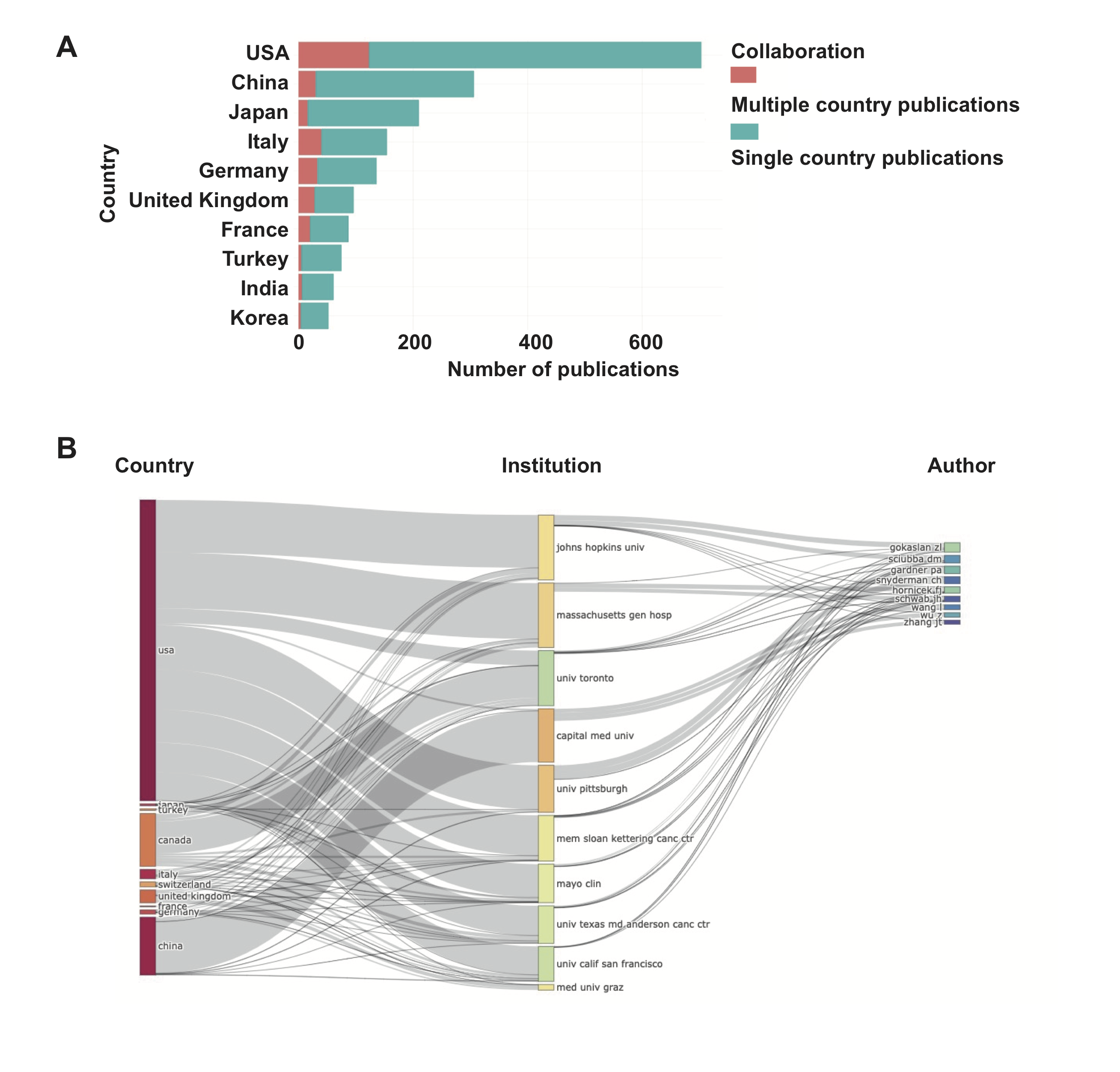

Supplement: Supplementary file 2 [file Image_2.tiff]

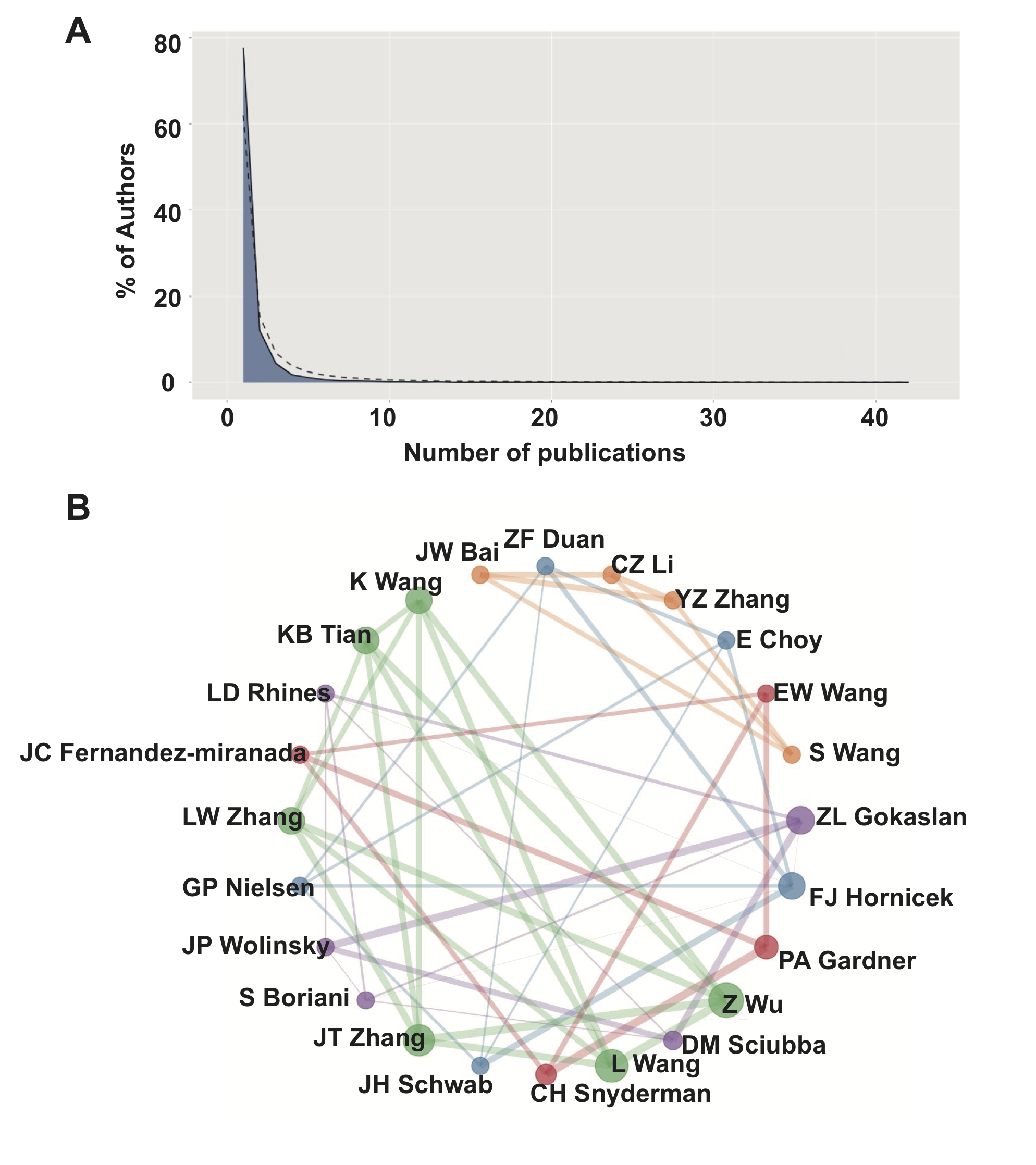

Supplement: Supplementary file 3 [file Image_3.tiff]

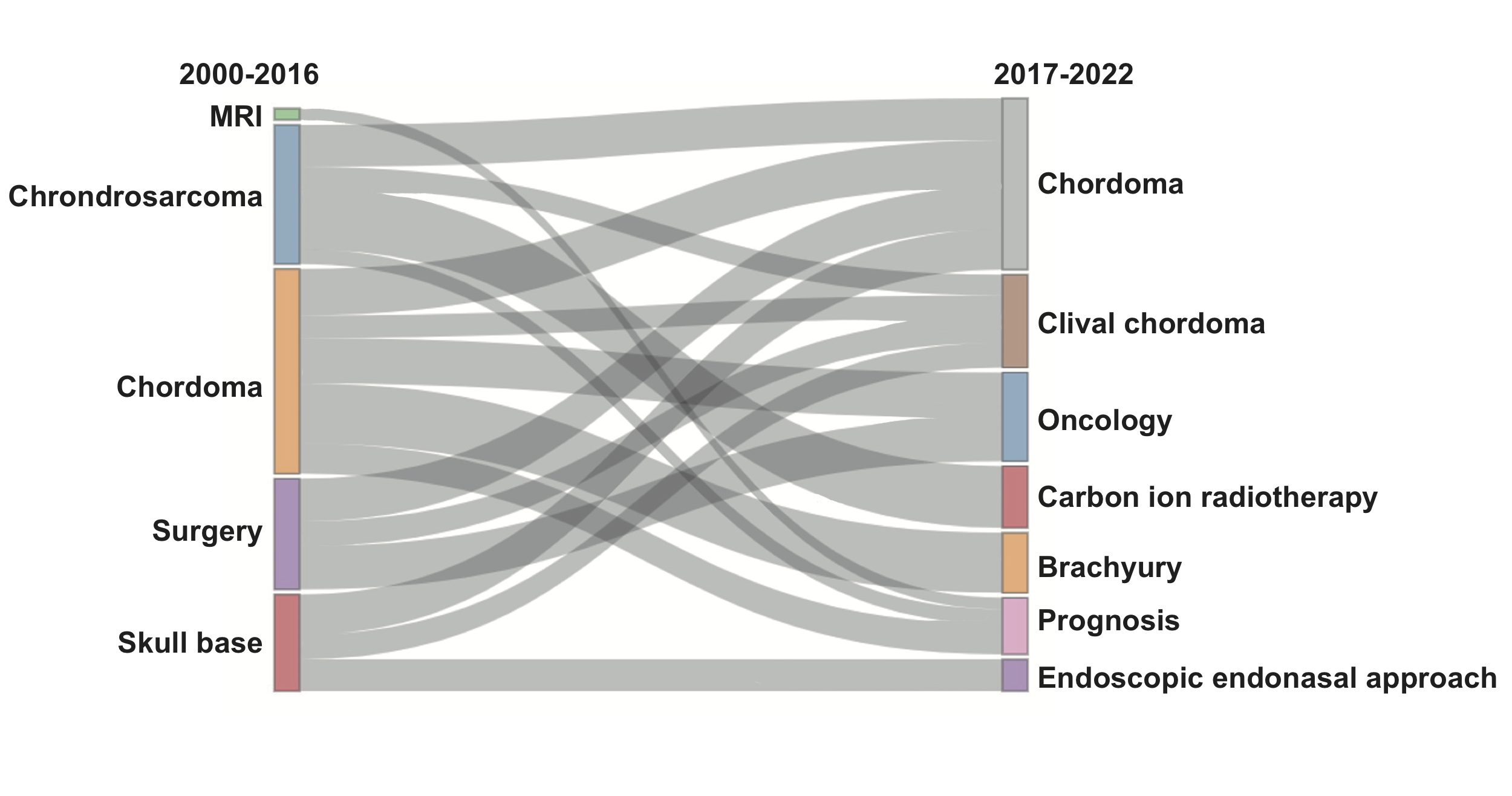

Supplement: Supplementary file 4 [file Image_4.tiff]

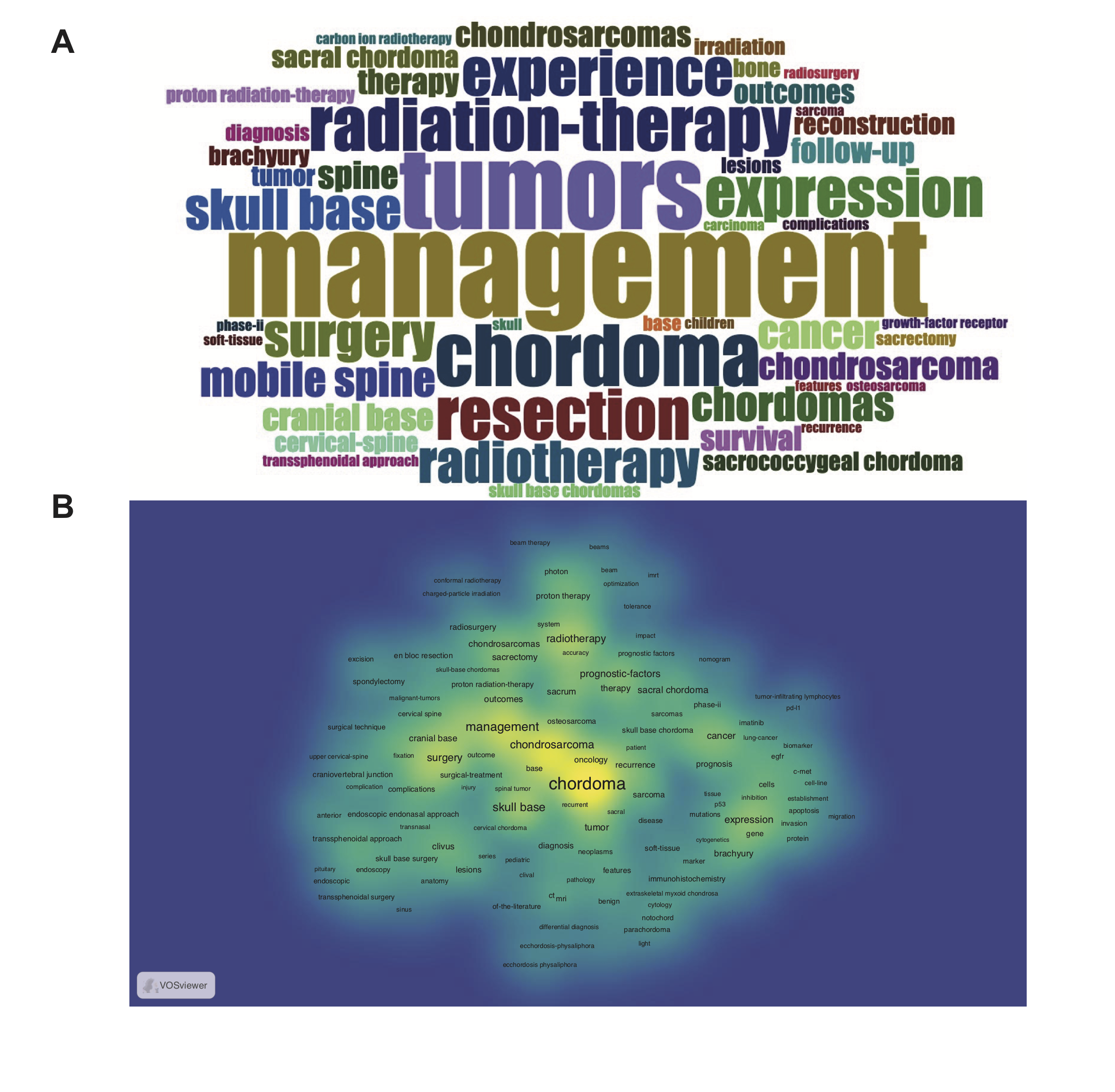

Supplement: Supplementary file 5 [file Image_5.tiff]
