# Supplementary material for: Research hotspots and trends of chordoma: A bibliometric analysis
Source: Front Oncol. 2022 Sep 16;12:946597. doi: 10.3389/fonc.2022.946597 (PMC9523362; doi:10.3389/fonc.2022.946597)
Supplement: Supplementary file 6 [file DataSheet_1.docx]

**Table S1. Top 10 most productive countries/regions in chordoma field.**

| Rank | Country/Region | Records | Total Citations | Citations per item |
| --- | --- | --- | --- | --- |
| 1 | USA | 703 | 17,509 | 24.91 |
| 2 | China | 306 | 3,002 | 9.81 |
| 3 | Japan | 210 | 4,029 | 19.19 |
| 4 | Italy | 154 | 3,104 | 20.16 |
| 5 | Germany | 136 | 3,523 | 25.90 |
| 6 | United Kingdom | 96 | 2,578 | 26.85 |
| 7 | France | 87 | 1,392 | 16.00 |
| 8 | Turkey | 75 | 868 | 11.57 |
| 9 | India | 61 | 418 | 6.85 |
| 10 | Korea | 52 | 551 | 10.60 |

**Table S2. Top 10 most productive institutions in chordoma field.**

| Rank | Institution | Country/Region | Records | Total Citations | h-index |
| --- | --- | --- | --- | --- | --- |
| 1 | Capital Medical University | China | 126 | 696 | 13 |
| 2 | University of Pittsburgh | USA | 124 | 2,555 | 24 |
| 3 | Massachusetts General Hospital | USA | 122 | 3,810 | 34 |
| 4 | Johns Hopkins University | USA | 116 | 2,565 | 30 |
| 5 | Medical University of Graz | Austria | 87 | 510 | 10 |
| 6 | Memorial Sloan Kettering Cancer Center | USA | 85 | 1,787 | 23 |
| 7 | Mayo Clinic | USA | 79 | 1,832 | 24 |
| 8 | University of Texas MD Anderson Cancer Center | USA | 71 | 2,064 | 26 |
| 9 | University of California, San Francisco | USA | 69 | 1,330 | 22 |
| 10 | University of Toronto | Canada | 66 | 1,029 | 17 |

**Table S3. Top 10 most contributing funds in chordoma field.**

| Rank | funds | Country/Region | records | Proportion (%) |
| --- | --- | --- | --- | --- |
| 1 | U.S. National Institutes of Health | USA | 142 | 6.21 |
| 2 | United States Department of Health and Human Services | USA | 142 | 6.21 |
| 3 | National Natural Science Foundation of China | China | 112 | 4.90 |
| 4 | National Cancer Institute | USA | 102 | 4.46 |
| 5 | Ministry of Education, Culture, Sports, Science and Technology | Japan | 26 | 1.14 |
| 6 | Chordoma Foundation | USA | 25 | 1.09 |
| 7 | European Commission | Europe | 23 | 1.01 |
| 8 | Japan Society for the Promotion of Science | Japan | 20 | 0.88 |
| 9 | Beijing Natural Science Foundation | China | 18 | 0.79 |
| 10 | Beijing Municipal Science and Technology Commission | China | 15 | 0.66 |

**Table S4. Top 10 highest impact factor journals in chordoma research**

| Rank | Journal | IF (2021) | Records | Total Citations | Citations per item |
| --- | --- | --- | --- | --- | --- |
| 1 | Nature Medicine | 53.440 | 1 | 55 | 55.00 |
| 2 | Nature Genetics | 38.330 | 2 | 261 | 130.50 |
| 3 | European Urology | 20.096 | 1 | 19 | 19.00 |
| 4 | Acta Neuropathologica | 17.088 | 5 | 198 | 39.60 |
| 5 | Journal of the American Chemical Society | 15.419 | 1 | 30 | 30.00 |
| 6 | Nature Communications | 14.919 | 3 | 98 | 32.67 |
| 7 | Science Advances | 14.136 | 1 | 68 | 68.00 |
| 8 | Journal for Immunotherapy of Cancer | 13.751 | 3 | 115 | 38.33 |
| 9 | Journal of Pineal Research | 13.007 | 1 | 12 | 12.00 |
| 10 | Cancer Research | 12.701 | 2 | 49 | 24.50 |

**Table S5. Top 10 most contributing authors in chordoma field**

| Rank | Author | Country | Records | Total citations | Average citation rate | h-index |
| --- | --- | --- | --- | --- | --- | --- |
| 1 | Junting Zhang | China | 21 | 269 | 12.81 | 9 |
| 2 | Huilin Yang | China | 20 | 364 | 18.20 | 10 |
| 3 | Yazhuo Zhang | China | 19 | 134 | 7.05 | 7 |
| 4 | Jianru Xiao | China | 18 | 196 | 10.89 | 8 |
| 5 | Kangwu Chen | China | 17 | 258 | 15.18 | 9 |
| 6 | Daniel M Sciubba | USA | 16 | 247 | 15.44 | 9 |
| 7 | Paul A Gardner | USA | 16 | 395 | 24.69 | 3 |
| 8 | Zhenfeng Duan | USA | 14 | 341 | 24.36 | 7 |
| 9 | Adrienne M Flanagan | United Kingdom | 14 | 1,125 | 80.36 | 7 |
| 10 | Liang Wang | China | 13 | 165 | 12.69 | 2 |

**Table S6. Top 20 most cited articles in chordoma sorted by total citations**

| Rank | Reference Styling | Title | Total citations | Average citations per year | DOI |
| --- | --- | --- | --- | --- | --- |
| 1 | McMaster et al., 2001 | Chordoma: incidence and survival patterns in the United States, 1973-1995 | 594 | 27.00 | 10.1023/a:1008947301735 |
| 2 | Yamasaki et al., 2005 | Apparent diffusion coefficient of human brain tumors at MR imaging | 353 | 19.61 | 10.1148/radiol.2353031338 |
| 3 | Vujovic et al., 2006 | Brachyury, a crucial regulator of notochordal development, is a novel biomarker for chordomas | 327 | 19.24 | 10.1002/path.1969 |
| 4 | Couldwell et al., 2004 | Variations on the standard transsphenoidal approach to the sellar region, with emphasis on the extended approaches and parasellar approaches: Surgical experience in 105 cases | 323 | 17.00 | 10.1227/01.neu.0000134287.19377.a2 |
| 5 | Antonescu et al., 2010 | EWSR1-POU5F1 Fusion in Soft Tissue Myoepithelial Tumors. A Molecular Analysis of Sixty-Six Cases, Including Soft Tissue, Bone, and Visceral Lesions, Showing Common Involvement of the EWSR1 gene | 307 | 23.62 | 10.1002/gcc.20819 |
| 6 | Boriani et al., 2006 | Chordoma of the mobile spine: Fifty years of experience | 273 | 16.06 | 10.1097/01.brs.0000200038.30869.27 |
| 7 | Fuchs et al., 2005 | Operative management of sacral chordoma | 267 | 14.83 | 10.2106/JBJS.D.02693 |
| 8 | Ryu et al., 2001 | Image-guided hypo-fractionated stereotactic radiosurgery to spinal lesions | 236 | 10.73 | 10.1097/00006123-200110000-00011 |
| 9 | Cappabianca et al., 2002 | Endoscopic endonasal transsphenoidal approach: Outcome analysis of 100 consecutive procedures | 223 | 10.62 | 10.1055/s-2002-36197 |
| 10 | Schwartz et al., 2008 | Endoscopic cranial base surgery: Classification of operative approaches | 216 | 14.40 | 10.1227/01.NEU.0000313231.81129.66 |
| 11 | Choi et al., 2008 | Identification of Nucleus Pulposus Precursor Cells and Notochordal Remnants in the Mouse: Implications for Disk Degeneration and Chordoma Formation | 209 | 13.93 | 10.1002/dvdy.21805 |
| 12 | Jho et al., 2001 | Endoscopic transsphenoidal surgery | 209 | 9.50 | 10.1023/A:1012969719503 |
| 13 | Schulz-Ertner., 2007 | Effectiveness of carbon ion radiotherapy in the treatment of skull-base chordomas | 208 | 13.00 | 10.1016/j.ijrobp.2006.12.059 |
| 14 | Stippler et al., 2009 | Endoscopic endonasal approach for clival chordomas | 199 | 14.21 | 10.1227/01.NEU.0000338071.01241.E2 |
| 15 | Paganetti et al., 2008 | Clinical implementation of full Monte Carlo dose calculation in proton beam therapy | 195 | 13.00 | 10.1088/0031-9155/53/17/023 |
| 16 | Casali et al., 2004 | Imatinib mesylate in chordoma | 191 | 10.05 | 10.1002/cncr.20618 |
| 17 | Fourney et al., 2005 | En bloc resection of primary sacral tumors: classification of surgical approaches and outcome | 185 | 10.28 | 10.3171/spi.2005.3.2.0111 |
| 18 | Yang et al., 2009 | T (brachyury) gene duplication confers major susceptibility to familial chordoma | 183 | 13.07 | 10.1038/ng.454 |
| 19 | Lomax et al., 2001 | Intensity modulated proton therapy: A clinical example | 183 | 8.32 | 10.1118/1.1350587 |
| 20 | Tzortzidis et al., 2006 | Patient outcome at long-term follow-up after aggressive microsurgical resection of cranial base chordomas | 171 | 10.06 | 10.1227/01.NEU.0000223441.51012.9D |
